# Seasonal and Soil Microbiota Effects on the Adaptive Strategies of Wild Goitered Gazelles Based on the Gut Microbiota

**DOI:** 10.3389/fmicb.2022.918090

**Published:** 2022-07-04

**Authors:** Wen Qin, Pengfei Song, Shoudong Zhang

**Affiliations:** ^1^State Key Laboratory of Plateau Ecology and Agriculture, Qinghai University, Xining, China; ^2^Northwest Institute of Plateau Biology, Chinese Academy of Sciences, Xining, China; ^3^Ministry of Education Key Laboratory for Biodiversity Science and Ecological Engineering, Coastal Ecosystems Research Station of the Yangtze River Estuary, School of Life Sciences, Fudan University, Shanghai, China; ^4^Rudi Drent Chair in Global Flyway Ecology, Conservation Ecology Group, Groningen Institute for Evolutionary Life Sciences (GELIFES), University of Groningen, Groningen, Netherlands

**Keywords:** *Gazella subgutturosa*, gut microbiota, soil microbiota, ecological process, adaptation

## Abstract

Seasonal variation in extreme environments is a threat to endangered species. The gut microbiota is important in the adaptive strategies of wild herbivores, and herbivores will contact the soil microbiota when they are feeding. However, there are no studies about the effects of soil microbiota on the gut microbiota of wild herbivores. Understanding the seasonal adaptive strategies of wild herbivores based on their gut microbiota and the effects of soil microbiota on the herbivorous gut microbiota is indispensable for making optimal conservation recommendations. To address those issues, we compared the diversity and functions of gut microbiota in goitered gazelles between winter and summer with a non-invasive fecal sampling method from the Qaidam Basin based on 16S rRNA V3–V4 regions. The data showed that seasonal variations caused the significant changes in gut microbiota at α-and β-diversity levels. The main gut microbial function was “Metabolism.” It showed significant seasonal changes. The goitered gazelles adapted to the seasonal changes by increasing the relative abundance of *Firmicutes*, *Christensenellaceae*, *Bacteroides* and the function about “Metabolism” in the winter to improve the adaptability. We also compared the effects of soil microbiota on the gut microbiota between winter and summer, covering source tracking analysis and the seasonal differences in ecological assembly processes. The contribution of soil microbiota on the gut microbiota of goitered gazelles was 5.3095% and 15.6347% in winter and summer, respectively, which was greater than on species of animals living underground. Seasonal variation also influenced the ecological processes of microbiota both in the gut and soil. Due to the differences in environments, the ecological processes between fecal microbiota and soil microbiota showed significant differences, and they were dominated by stochastic processes and deterministic processes, respectively. The soil microbiota has contributed to the gut microbiota, but not a decisive factor. Our research laid the foundation on the seasonal and soil microbiota effects on the adaptive strategies of goitered gazelles, and is the first study to explain the soil microbiota influence on the gut microbiota of wild herbivores.

## Introduction

Gut microbiota plays an essential role in the health and adaptation of the herbivores (Ding et al., 2018; Wu et al., 2019; Cui et al., 2020). Seasonal variation is the main factor causing changes in gut microbiota. Herbivores in extreme environments have evolved seasonal adaptive strategies based on their gut microbiota (Guo et al., 2021). Understanding the relationship between host adaptability and gut microbial seasonal variation is important for managing the conservation of wild herbivores in extreme environments. The significant seasonal changes in the external environment can change the gut microbial diversity (Ren et al., 2017; Smits et al., 2017). For example, diet is a major factor that affects seasonal variations in the gut. Seasonal dietary changes may be related to the different functions of gut microbiota between the wet and dry seasons in the Hadza (Smits et al., 2017). Under the same feeding conditions and environment, Sprague–Dawley male rats showed seasonal gut microbial variations (Liu et al., 2018). Gut microbiota seasonal changes can help the host adapt to environmental changes. Seasonal variation in gut microbiota may help the wild Tibetan macaque adapt to diet changes and provide sufficient energy for them in extreme climates. In winter, the increase in the relative abundance of *Proteobacteria* and *Succinivibrio* of the wild Tibetan macaque is associated with energy accumulation and utilization (Sun et al., 2016). *Treponema*, whose relative abundance is higher in winter, can help the yak degrade plant polysaccharides from hay or from a concentrated diet. The relative abundance of *Butyrivibrio_2* is higher in summer, which is of benefit for the yak to degrade complex carbohydrates and protein (Ma et al., 2019). Seasonal changes are closely related to changes in gut microbial diversity, and as a result, changes in gut microbiota can be used as indicators to reflect the adaptability of the host (Bergmann et al., 2015; Xu et al., 2015; Xue et al., 2015; Chen et al., 2017; Hicks et al., 2018).

The goitered gazelle, *G. subgutturosa* (Güldenstaedt, 1780), is mainly distributed in arid and subarid areas, including shrubland, grassland and desert. There are fewer than 49,000 individuals of goitered gazelle, and it is listed as a vulnerable species based on IUCN red list. Climate change and temperature extremes are among its main threats (IUCN SSC Antelope Specialist Group, 2017). In the Qinghai-Tibet plateau, goitered gazelles are mainly found in the Qaidam Basin. The goitered gazelle is the representative herbivore of the Qaidam Basin and is the foundation for the ecological balance of this area. The Qaidam Basin is located in the eastern portion of the Qinghai-Tibet plateau. The climate is characterized by evaporation greater than rainfall, cold and long winters and hot and dry summers, the altitude ranges from 2,600 to 5,500 m (Shen, 1998; Shi et al., 2005; Wei et al., 2014; Li et al., 2017a). In this location, goitered gazelles must adapt to large seasonal temperature differences and drought. Goitered gazelles can adapt to the environment seasonal variations by adjusting water evaporation and the size of organs, including the heart, liver and brain (Ostrowski et al., 2006). Goitered gazelle is a representative ungulate species that can be used to study adaptations to arid environment. However, prior studies on the adaptability of goitered gazelles have mainly focused on ecological adaptation and behavioral characteristics (Blank et al., 2012, 2015; Zhang et al., 2021). The main research sites have been in Middle Asia and the Xinjiang Uygur Autonomous Regions. There have been no systematic studies on the adaptation of goitered gazelle to the seasonal changes based on gut microbiota in the extreme environment of the Qaidam Basin. Therefore, understanding those issues is the basis for the conservation of goitered gazelles in the Qaidam Basin and also an urgent problem to be solved now.

The environment of Keke Town in Qinghai Province has the universal characters of the Qaidam Basin environment, and it is a representative site of the Qaidam Basin. The goitered gazelle population in Keke Town is large, and it is a major population within the Qaidam Basin. The gazelles in this population do not migrate and are present in both summer and winter. Some goitered gazelle populations in Xinjiang embark on long-distance migration to cope with seasonal environmental variation (Sun et al., 2002), but we have not observed the population in Keke Town migrating over long distances. The goitered gazelle population in Keke town may have a different strategy to adapt to seasonal variation. It is unknown how the Keke population has adapted to the seasonal variations in its environment. We believe that a study on the seasonal variations in gut microbiota of the goitered gazelle population in Keke Town is an representativeness for studying the seasonal adaptation strategies of wild herbivores in extreme environments. This research expands knowledge of both this population and the entire species.

The goitered gazelle mainly feeds on *Chenopodiaceae* and *Gramineae* (Xu et al., 2008), so goitered gazelles are likely in contact with the surface of the soil when they are feeding. During feeding, the gazelle may ingest microorganisms from the soil. More attention is given to the direct impact of climate change on wildlife, but herbivores frequently contact the soil when eating, and soil is also a factor that can influence changes in herbivorous gut microbiota. There is no known analysis on the source tracking between the gut microbiota of herbivores and soil microbiota. Microbial diversity in soil shows different characters and seasonal changes, and seasonal changes in soil microbiota may benefit the survival and adaptation of associated animals (Garcia-Alvarez and Ibañez, 1994; Guimaraes et al., 2020; Ishaq et al., 2020). For example, *Gemmatimonadetes* in soil has seasonal variations and uses these to adapt to changes in the soil environment (DeBruyn et al., 2011). Soil may also explain the gut microbiological properties of the host (Grieneisen et al., 2019; Hannula et al., 2019). However, most studies on seasonal changes of soil microbiota are related to crops and climate changes, and few focus on animal conservation.

The ecological aspects of communities are important in revealing the process of community formation, including the microbial community of soil and the animal gut. Deterministic and stochastic processes are the dominant components of community formation. Deterministic processes involve biotic and abiotic factors, and stochastic processes involve factors under which all species are ecologically equivalent (Mo et al., 2021). The dominant causes (deterministic or stochastic processes) in the assembly process of gut microbiota from different wild animals are changed in different environments (Li et al., 2019b, 2022). However, there are few studies about the ecological assembly process of gut microbiota, not to mention their effect on the host of gut microbiota. This gap needs to be filled. We speculated that the gut microbial formation process of goitered gazelles in Keke Town may show seasonal variations to adapt to the seasonal changes in the Qaidam Basin.

In this study, we set out to solve two scientific problems. The first one is what are the seasonal changes of gut microbiota in goitered gazelles, and how do goitered gazelles adapt to environmental seasonal changes based on gut microbiota? The second one is the effects of soil microbiota on seasonal adaptation of gut microbiota in goitered gazelles. Here, we collected 78 fecal samples from goitered gazelles from Qaidam Basin, using a non-invasive method. We used 16S rRNA V3-V4 regions to compare the differences of microbiota in feces and soils between winter and summer and describe the seasonal variations and the effects of soil microbiota on fecal microbiota of goitered gazelle. This study describes the seasonal adaptability of goitered gazelle populations in Qaidam Basin, and the data are beneficial to the conservation and management of this species.

## Materials and Methods

### Ethics Statement

All experiments, including the sample collection methods, followed the principles of the Ethical Committee for Experimental Animal Welfare of the Northwest Institute of Plateau Biology.

### Sample Collection

All the samples were collected in Keke Town of Wulan County, Qinghai Province, China (36.97°N 98.04°E). In the winter, a total of 47 fresh fecal pellets of goitered gazelles (one per individual) and 8 soil samples were collected on 3–4 December 2020. In the summer, a total of 31 fresh fecal pellets from goitered gazelles (one per individual) and 6 soil samples were collected on 12 August 2021.

**Figure 1:**
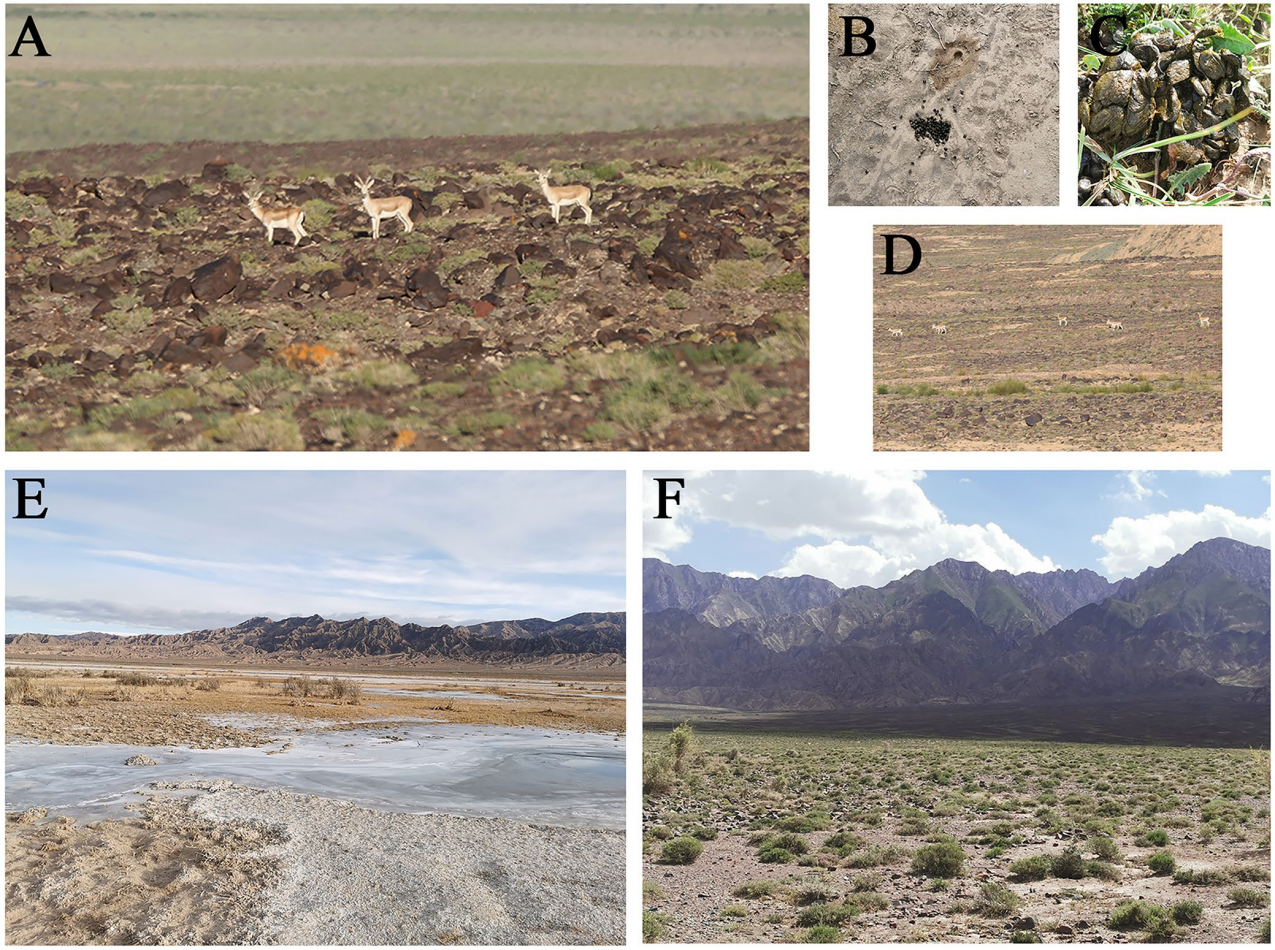
**(A)** The picture of goitered gazelles in Keke Town; **(B)** The fresh feces of one individual of goitered gazelle in the winter; **(C)** The fresh feces of one individual of goitered gazelle in the summer; **(D)** The picture of a group of goitered gazelles in Keke Town; **(E)** The sampling area of Keke Town in the winter; **(F)** The sampling area of Keke Town in the summer.

According to our investigation, the goitered gazelles in Keke Town usually go to the lake to drink water after sunrise and mostly defecate near the lake. So our sampling time was after sunrise and before noon. The goitered gazelles’ fecal pellets were concentrated rather than scattered on the ground, allowing them to be identified as individuals rather than groups. In the winter, fresh fecal pellets were either moist and warm, or they had a frosty surface but were moist on the inside. In the summer, fresh fecal pellets were either moist and warm, or slightly dry on the surface but moist on the inside (Figure 1). We tried to select inside fecal pellets that were moist and not stuck to the soil, and we collected at least three fecal pellets (more than 0.5 g) from each individual. Each individual fresh fecal sample was collected with disposable polyethylene (PE) gloves to avoid contamination. Individual fecal samples were placed in ziplock bags (one per individual), labeled with water-proof and alcohol-proof label pen, and then stored in liquid nitrogen for less than 14 days.

During soil sampling, we first collected topsoil from three different sites; the distance between them was about 100 m. Then we mixed the three topsoils and passed them through a 100 mesh sieve to remove rocks and dead plant material. Finally, what we had was 1 soil sample. We repeated the above steps for each soil sample. To make the soil samples representative, the distance between each soil sample we obtained was more than 500 m. All the soil samples were labeled, stored in cryopreservation tubes and stored in liquid nitrogen for fewer than 14 days. Prolonged storage of fecal samples and soil samples was at −80°C.

### DNA Extraction, Amplification, and Sequencing

Library preparation and sequencing were performed at the Majorbio Bio-Pharm Technology Co. Ltd. (Shanghai, China). Total DNA was extracted with the E.Z.N.A.^®^ soil DNA Kit (Omega Bio-tek, Norcross, GA, United States) using manufacturer instructions. All of the DNA samples were qualified and determined with a NanoDrop 2000 UV–vis spectrophotometer (Thermo Scientific, Wilmington, DE, United States). The 16S rRNA V3-V4 regions were amplified with primers 338F(5′-ACTCCTACGGGAGGCAGCAG-3′) and 806R(5′-GGACTACHVGGGTWTCTAAT-3′). PCR reactions were performed in 20 μl and included 4 μl of 5× TransStart FastPfu buffer (Thermo Fisher Scientific, Wilmington, DE, United States), 0.4 μl of TransStart FastPfu DNA Polymerase (Thermo Fisher Scientific, Wilmington, DE, United States), 10 ng of extracted DNA as template, 0.8 μl of 5 μM each primer, 2 μl of 2.5 mM deoxynucleoside triphosphates (dNTPs) and additional ddH_2_O up to 20 μl. The PCR protocol was: 30 s at 95°C, 30 s at 55°C, and 45 s at 72°C for 27 cycles. All PCR products were mixed and assessed by 2% agarose gel electrophoresis, purified with the AxyPrep DNA Gel Extraction Kit (Axygen Biosciences, Union City, CA, United States) based on manufacturer instructions. The purified amplicons were sequenced on the Illumina MiSeq sequencing platform (Illumina, San Diego, United States). A total of 92 samples underwent this analysis. The raw data are available from the Sequence Read Archive (SRA) under the accession number: PRJNA 825477.

### The Bioinformatics Pipeline

The bioinformatics pipeline was mainly conducted in QIIME2 (Bolyen et al., 2019). In brief, after demultiplexed according to the specific barcode sequences of each sample, the resulting sequences were merged using FLASH (v1.2.11) software (Magoč and Salzberg, 2011) and quality filtered with fastp (0.19.6; Chen et al., 2018). The DADA2 (*via* q2-dada2 plugin) was used to denoise the sequences with recommended parameters (Callahan et al., 2016) to obtain raw amplicon sequence variant (ASV) table and raw ASV representative sequences.

Based on the curated SILVA SSU NR99 (version 138) database,^1^ the reference sequence annotation and curation pipeline (RESCRIPt) were used to prepare a QIIME2 compatible amplicon-specific naïve Bayes classifier to improve the quality of classification (Robeson et al., 2021), following the protocol suggested by the author.^2^ Taxonomic classification was performed with Q2-feature-classifier plugin (0.8 confidence). The taxonomy-based filtering was used to remove the ASVs that belong to mitochondria, chloroplast, or archaea. ASVs with relative abundance lower than 0.01% as well as present in fewer than five samples were also excluded. The ASV table was than rarefied to the minimum sequencing depth of all samples and used for further analyses except FEAST analysis, which required a raw ASV table.

The Venn diagram was analyzed using package “stats” (R Core Team, 2019). The pairwise comparisons of microbiota between gut and soil at the phylum level, family level, and genus level were all calculated based on the Wilcoxon rank-sum test. Alpha diversity indexes (Shannon and Simpson) were calculated using the package “vegan” (Oksanen et al., 2019), and compared by Wilcoxon rank-sum test between any two groups using package “stats” (R Core Team, 2019). All distance-based analyses were performed based on the respective Bray-Curtis distances, which was calculated by package “vegan” (Oksanen et al., 2019). The non-metric multidimensional scaling (NMDS), analysis of similarities (ANOSIM) and permutational multivariate analysis of variance (PERMANOVA) were performed using the package “vegan” with 999 permutations (Oksanen et al., 2019), and visualized with the package “ggplot2” (Wickham, 2016). LefSe (linear discriminant analysis effect size) analysis was performed using LefSe software (LDA score = 4; Segata et al., 2011). All the relevant bioinformatic analyses were performed on the free online platform of Majorbio Cloud Platform (Shanghai Majorbio Bio-pharm Technology Co., Ltd., Shanghai, China).

### Source Tracking Analysis

We used the fast expectation–maximization microbial source tracking (FEAST) to reveal the origins of the fecal microbiota of goitered gazelles (Shenhav et al., 2019). We considered the fecal microbiota of every sample as a sink, and the soil microbiota of each corresponding sampling site were considered a source. Fecal microbiota in each season were source tracked to soil microbiota in the corresponding season. Taxa that could not be classified to the input sources were categorized as unknowns. The parameters were COVERAGE = 12,234, EM_iterations = 10,000,000 in both two FEAST analysis.

### The Ecological Assembly Process of Microbiota in Gut and Soil

We estimated contributions of the stochastic and deterministic assembly processes in the microbiota of gut and soil with the modified stochasticity ratio (MST). MST values > 0.5 and < 0.5 indicated that the dominant assembly processes were deterministic process and stochastic process, respectively (Ning et al., 2019). The MST were calculated using “NST” (normalized stochasticity ratio) package with 30,000 simulations in R and Rstudio (Zhou et al., 2014; Ning et al., 2019) followed the protocol that author suggested.

With the neutral community model, we assessed the effects of stochastic processes on the microbiota of gut and soil and evaluated the goodness of fit to the model by non-linear least-squares (Sloan et al., 2006; Östman et al., 2010). This model was performed with the package “Minpack.lm” in R and Rstudio.^3^ The R^2^ values were <1. When the R^2^ values increased, the predominance of the stochastic process was also greatly increased.

To test the clustering or overdispersion of microbiota in gut and soil, we examined the deviation of each observed metric from the average of the null model [checkerboard score (C-score); Stone and Roberts, 1990]. The standardized effect size (SES) included standardized values to comparisons among assemblages and was calculated under the null model. The C-score was assessed based on 30,000 simulations by the sequential swap randomization algorithm with the “EcoSimR” package in R and Rstudio^4^ (Stone and Roberts, 1990; Mo et al., 2021). The values of SES indicate the strength of the effect of deterministic processes on the assemblage (Li et al., 2017b; Mo et al., 2021).

## Results

### Comparison of Gut Microbial Diversity in Goitered Gazelles Between Winter and Summer

**Figure 2:**
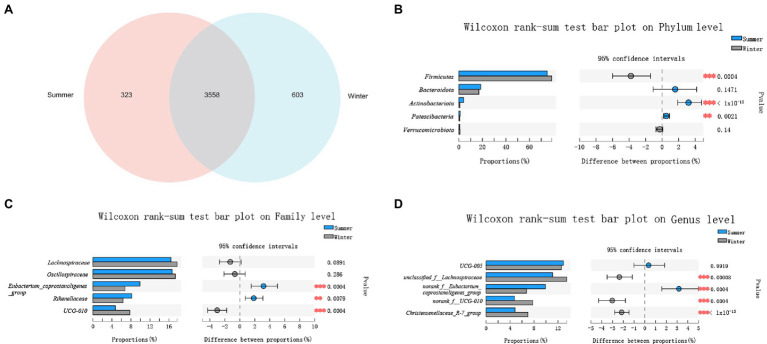
Comparison of gut microbial diversity between winter and summer. **(A)** A Venn diagram at amplicon sequence variant (ASV) level; **(B)** Top 5 phyla; **(C)** Top 5 families; **(D)** Top 5 genera. Significant differences in gut microbiota between winter and summer are indicated with value of *p* and asterisks (** if 0.001 < *p* < 0.01 and *** if *p* < 0.001).

There were 3,558 ASVs shared between winter and summer, and there were more unique ASVs in the winter (603 ASVs). In the summer, there were 323 unique ASVs (Figure 2A).

In the winter and summer, the top 5 phyla in relative abundance were *Firmicutes* (78.95% ± 4.66%, 75.15% ± 5.51%), *Bacteroidota* (17.02% ± 4.60%, 18.60% ± 6.49%), *Actinobacteriota* (0.83% ± 0.79%, 4.03% ± 3.93%), *Patescibacteria* (0.63% ± 0.44%, 1.13% ± 1.0%), *Verrucomicrobiota* (0.96% ± 1.10%, 0.66% ± 0.71%). Only the relative abundance of *Bacteroidota* and *Verrucomicrobiota* showed no significant seasonal differences (*p* > 0.05; Figure 2B).

At family level, the top 5 families in relative abundance were *Oscillospiraceae* (17.34% ± 2.30%, 16.67% ± 3.54%), *Lachnospiraceae* (17.70% ± 2.68%, 16.42% ± 3.61%), *Eubacterium_coprostanoligenes_group* (6.74% ± 1.43%, 9.90% ± 4.74%), *Rikenellaceae* (6.32% ± 1.57%, 8.18% ± 3.23%), *UCG-010* (7.77% ± 3.93%, 4.74% ± 1.80%). Only the relative abundance of *Lachnospiraceae* and *Oscillospiraceae* showed no significant differences between winter and summer (*p* > 0.05; Figure 2C).

At genus level, the top 5 bacteria in relative abundance were *UCG-005* (12.56% ± 2.38%, 12.91% ± 3.41%), *unclassified_f__Lachnospiraceae* (13.47% ± 2.16%, 11.10% ± 2.80%), *norank_f__Eubacterium_coprostanoligenes_group* (6.74 %± 1.43%, 9.90% ± 4.75%), *Christensenellaceae_R-7_group* (6.99% ± 1.31%, 4.83 %± 1.58%) and *norank_f__UCG-010* (7.77% ± 3.93%, 4.74% ± 1.80%). Only the relative abundance of *UCG-005* showed no significant differences between winter and summer (*p* > 0.05; Figure 2D).

The relative abundance of *Firmicutes* was significantly higher in the winter (*p* < 0.05), and the *Bacteroidota* was higher in the summer, showed no significant differences between winter and summer (*p* > 0.05). The ratio of *Firmicutes*/*Bacteroidota* was higher in the summer but there was no significant differences between summer and winter (*p* > 0.05).

**Figure 3:**
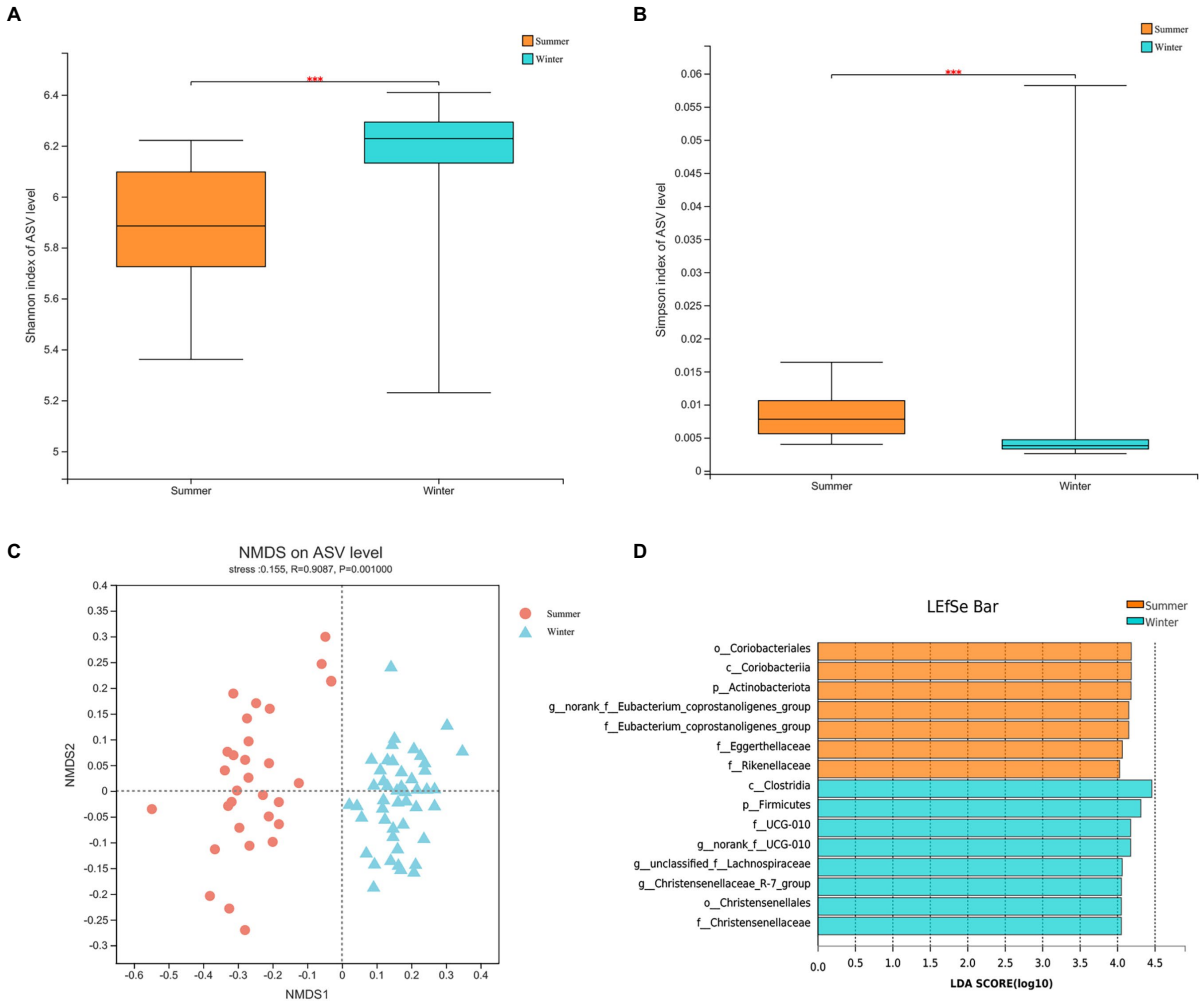
The α-diversity between winter and summer in gut microbiota: **(A)** Shannon index at amplicon sequence variant (ASV) level; **(B)** Simpson index at ASV level; **(C)** The non-metric multidimensional scaling (NMDS) analysis of gut microbiota between winter and summer; **(D)** The biomarkers of gut microbiota based on linear discriminant analysis effect size (LEFSe) analysis between winter and summer from phylum to genus.

At α-diversity level, based on Shannon index (Winter = 6.1904 ± 0.1859; Summer = 5.8871 ± 0.2335) and Simpson index (Winter = 0.0052 ± 0.0080; Summer = 0.0084 ± 0.0035), the α-diversity of gut microbiota in the winter was significantly higher (*p* < 0.05; Figures 3A,B).

At β-diversity, the gut microbial diversities between winter and summer showed significant differences based on PERMANOVA (Permutational MANOVA) analysis (R^2^ = 0.1261; *p* < 0.05; Appendix 1). The Anosim analysis at the ASV level (R = 0.9087, *p* = 0.001) supports this result. The NMDS analysis also showed that there was obvious separation in gut microbiota between winter and summer (Figure 3C). The LEFSe (Linear discriminant analysis Effect Size) analysis indicates that a total of 15 bacteria are biomarkers, including *Firmicutes*, *Actinobacteriota*, *Eubacterium_coprostanoligenes_group*, *Christensenellaceae* and *Christensenellaceae_R-7_group* (Figure 3D).

### Differences of Gut Microbial Functions in Goitered Gazelles Between Winter and Summer

The main function is “Metabolism” both in the winter and summer and the relative abundance are all above 58%. The following are functions of “Environmental Information Processing” and “Genetic Information Processing” based on KEGG database with Tax4Fun. The relative abundance of functions about “Metabolism” and “Cellular Processes” is significantly higher in the winter, and that of “Genetic Information Processing” is significantly lower in the winter (*p* < 0.05). There were no significant differences in functions about “Environmental Information Processing” between winter and summer (*p* > 0.05; Table 1).

**Table 1:** The comparison of relative abundance of functions in gut microbiota between winter and summer at level 1 based on KEGG database.

Pathway level 1	Cellular processes	Environmental information processing	Genetic information processing	Human diseases	Metabolism	Organismal systems
Winter	3.17%	19.35%	13.97%	2.41%	60.05%	0.91%
Summer	1.88%	19.47%	17.01%	2.38%	58.02%	1.14%
Value of *p*	2.03 × 10^−11^	0.357431	7.85 × 10^−11^	0.5239	6.24 × 10^−12^	7.06 × 10^−12^

At pathway level 3, the main function was “ABC transporters,” whose relative abundance was all above 10% in the two seasons. The following are “Two-component system” (ko02020), “Aminoacyl-tRNA biosynthesis” (ko00970), and “Purine metabolism” (ko00230), whose relative abundance were greater than 3% in both seasons. All the top 10 functions showed significant differences between winter and summer. Only the relative abundance of “Two-component system” was significantly higher in the winter (*p* < 0.05; Table 2).

**Table 2:** The comparison of relative abundance of functions in gut microbiota between winter and summer at level 3 based on KEGG database.

Pathway level 3	Description	Winter	Summer	Value of *p*
ko02010	ABC transporters	10.84%	11.94%	1.08 × 10^−7^
ko00970	Aminoacyl-tRNA biosynthesis	3.40%	4.46%	1.63 × 10^−11^
ko00230	Purine metabolism	3.74%	4.43%	1.29 × 10–^11^
ko02020	Two-component system	5.51%	4.23%	1.71 × 10^−10^
ko00240	Pyrimidine metabolism	2.76%	3.44%	6.52 × 10^−11^
ko03010	Ribosome	2.11%	2.71%	4.15 × 10^−11^
ko00550	Peptidoglycan biosynthesis	2.04%	2.61%	9.34 × 10^−11^
ko00500	Starch and sucrose metabolism	2.02%	2.58%	3.88 × 10^−10^
ko00520	Amino sugar and nucleotide sugar metabolism	2.20%	2.48%	8.84 × 10^−7^
ko02060	Phosphotransferase system (PTS)	2.03%	2.34%	0.0007

### Taxonomic Differences of Soil Microbial Diversity Between Winter and Summer

**Figure 4:**
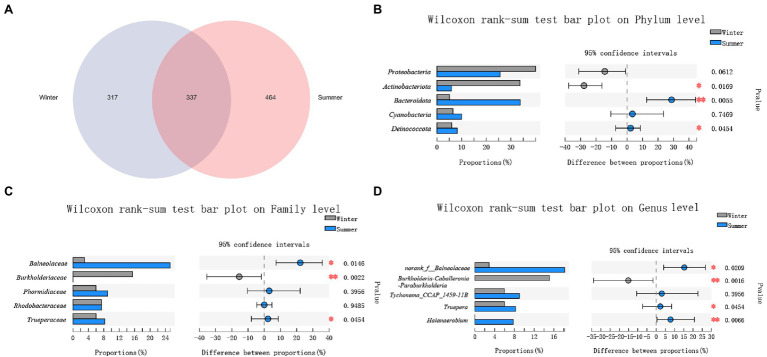
Comparison of soil microbial diversity between winter and summer. **(A)** A Venn diagram at amplicon sequence variant (ASV) level; **(B)** Top 5 phyla; **(C)** Top 5 families; **(D)** Top 5 genera. Significant differences in soil microbiota between winter and summer are indicated with value of *p* and asterisks (* if 0.01 < *p* < 0.05 and ** if 0.001 < *p* < 0.01).

Between winter and summer, a total of 337 ASVs were shared in soil microbiota. There were more unique ASVs (464) in summer, and only 317 unique ASVs present in the winter. This is the opposite of the results seen in the fecal microbiota (Figure 4A).

The top 5 bacteria were *Actinobacteriota* (33.68% ± 16.79%, 5.87% ± 2.41%), *Deinococcota* (5.99% ± 12.57%, 8.25% ± 3.10%), *Proteobacteria* (39.97% ± 17.16%, 25.60% ± 12.06%), *Bacteroidota* (5.06% ± 8.67%, 33.80% ± 20.26%), *Cyanobacteria* (6.50% ± 9.77%%, 9.97 ± 21.28%) at phylum level between winter and summer. Only the relative abundance of *Proteobacteria* and *Cyanobacteria* showed no significant differences between winter and summer (*p* > 0.05; Figure 4B).

At family level, the top 4 families were *Balneolaceae* (2.98% ± 8.22%, 25.21% ± 18.01%), *Burkholderiaceae* (15.45% ± 27.68%, 0.02% ± 0.05%), *Rhodobacteraceae* (7.39% ± 5.12%, 7.39% ± 4.33%), *Trueperaceae* (5.99% ± 12.57%， 8.25% ± 3.10%), *Phormidiaceae* (5.97% ± 9.29%， 9.01% ± 21.29%) between winter and summer. Only the relative abundance of *Phormidiaceae* and *Rhodobacteraceae* showed no seasonal significant differences (*p* > 0.05; Figure 4C).

At genus level, the top 5 genera were *norank_f__Balneolaceae* (2.88% ± 8.03%, 18.18% ± 13.97%), *Burkholderia-Caballeronia-Paraburkholderia* (15.12% ± 27.10%, 0), *Truepera* (5.99% ± 12.57%, 8.25% ± 3.10%), *Tychonema_CCAP_1459-11B* (5.97% ± 9.29%, 9.01% ± 21.29%), *Halanaerobium* (0.01% ± 0.02%, 7.78% ± 15.51%) between winter and summer. Only the relative abundance of *Tychonema_CCAP_1459-11B* showed no significant seasonal differences (*p* > 0.05; Figure 4D).

Among these bacteria from phylum to genus, only the relative abundance of *Actinobacteriota*, *Burkholderiaceae* and *Burkholderia-Caballeronia-Paraburkholderia* were significantly higher in the winter (*p* < 0.05; Figures 4B–D).

**Figure 5:**
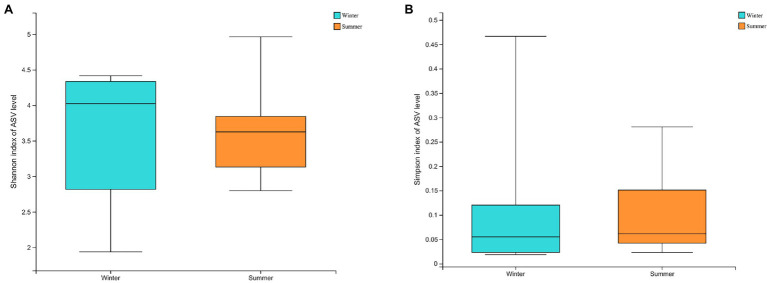
The α-diversity between winter and summer in soil microbiota: **(A)** Shannon index at amplicon sequence variant (ASV) level; **(B)** Simpson index at ASV level.

The α-diversity of soil microbiota was higher in the summer based on Shannon index (Winter = 3.5721 ± 0.9662; Summer = 3.6492 ± 0.7680), and Simpson index (Winter = 0.1137 ± 0.1515; Summer = 0.1072 ± 0.1010), there were no significant differences present (*p* > 0.05; Figures 5A,B).

At β-diversity, based on PERMANOVA analysis, the diversity of soil microbiota showed significant differences between winter and summer at ASV level (R^2^ = 0.3059; *p* < 0.05; Appendix 2). The Anosim analysis also indicated that inter-groups differences were greater than intra-groups differences (R = 0.6085; *p* = 0.0040) at the ASV level.

### Source Tracking Analysis of Fecal Microbiota From Soil Microbiota

The total contribution ratio of soil microbiota to fecal microbiota in the winter ranged from 4.12% to 6.84%, and the average contribution was ~5.31%. In the summer, the soil microbiota contributed more to the gut microbiota of goitered gazelle. The contribution rate ranged from 10.35% to 25.56% and the average value was 15.63%. Based on a *t*-test, there was a significant difference in soil microbiota contribution between winter and summer (*p* < 0.05).

### Deterministic and Stochastic Processes in the Gut and Soil

**Figure 6:**
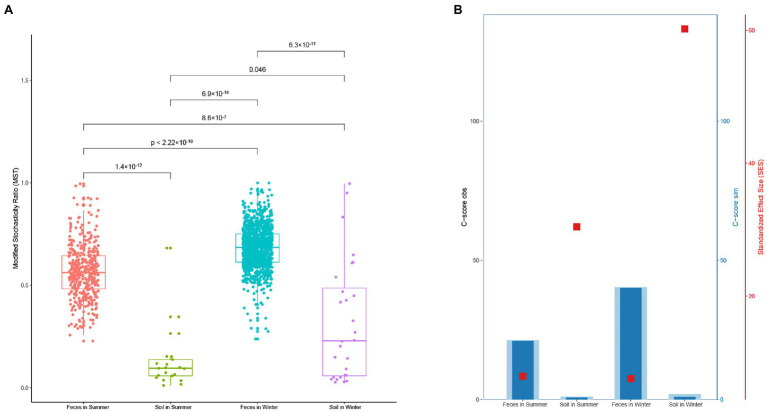
**(A)** Modified stochasticity ratio (MST) analysis of microbiota in gut and soil between winter and summer. **(B)** Checkerboard score (C-score) of microbiota in gut and soil between winter and summer based on the null model.

The MST distribution in fecal microbiota from the winter and summer both exceeded the threshold value (0.5). This suggests that the dominant ecological process in fecal microbiota is a stochastic process. The MST values of soil microbiota from the winter and summer were both less than 0.5, indicating that the dominant ecological process is deterministic (Figure 5). The C-score results also revealed that soil microbiota in the winter showed the highest standardized effect size (SES), followed by soil microbiota in the summer, fecal microbiota in the summer and fecal microbiota in the winter (Figure 6).

**Figure 7:**
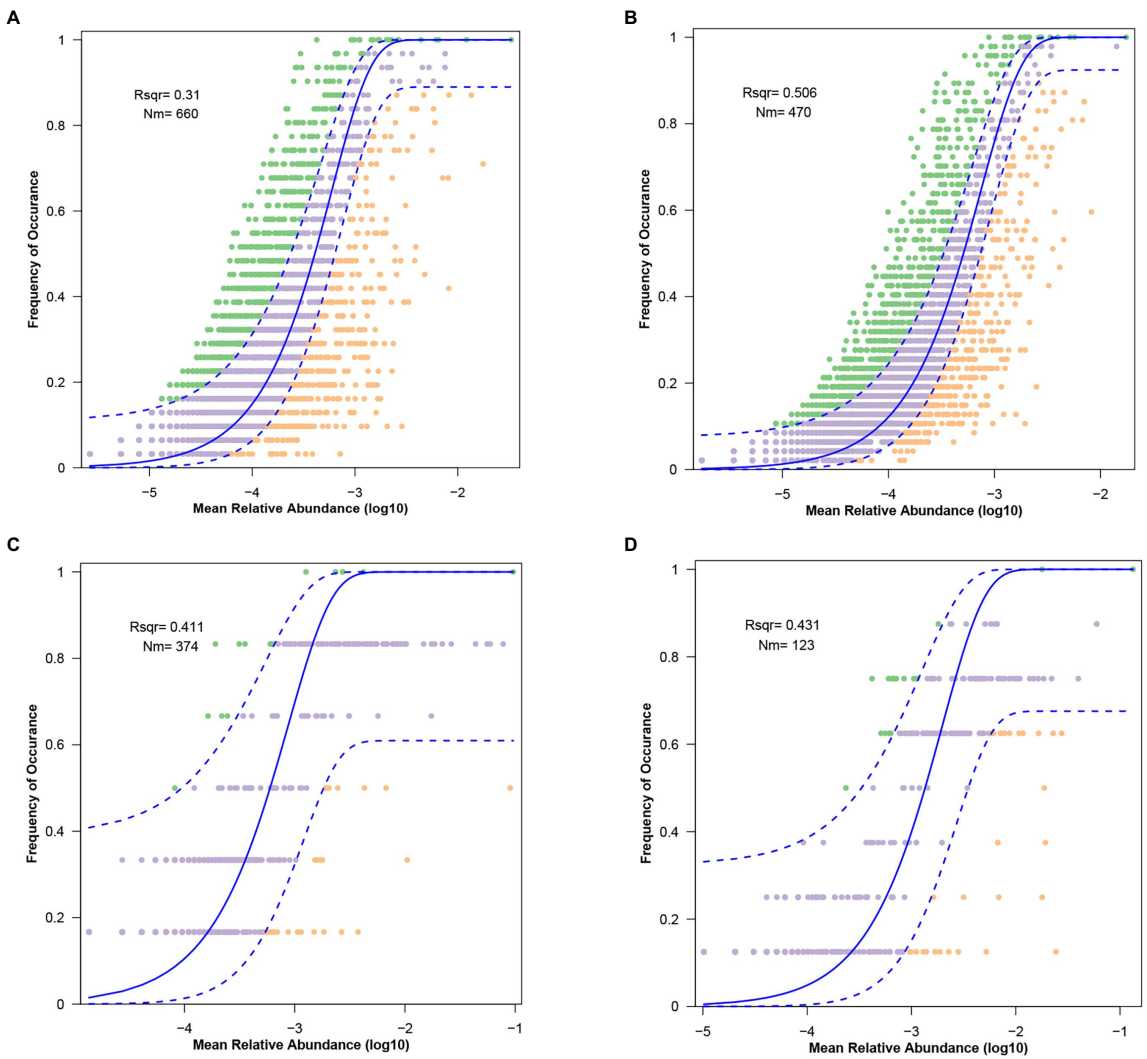
The predicted occurrence frequencies for **(A)** gut microbiota from summer, **(B)** gut microbiota from winter, **(C)** soil microbiota from summer, and **(B)** soil microbiota from winter. The solid blue line indicates the best fit to the neutral community model (NCM), the dashed blue line means 95% confidence intervals around the NCM prediction.

A higher SES value indicates a stronger deterministic process, and this result means the strongest deterministic process occurs in the winter soil microbiota. The neutral community model (NCM) showed that the microbiota in the gut and soil are weakly influenced by stochastic processes. The values of R^2^ decreased from winter to summer and the values of Nm increased from winter to summer, which is associated with diffusivity (Figures 7A–D).

## Discussion

### Seasonal Adaptation Strategies of Goitered Gazelles Based on Gut Microbiota

Between winter and summer, the gut microbial diversity showed significant differences. This result was consistent with other wildlife studies including musk deer and white-lipped deer on the Qinghai-Tibetan plateau (Jiang et al., 2021; Li et al., 2022). For herbivores in extreme environments, seasonal variation in gut microbiota is an essential adaptive strategy. It allows for the efficient digestion of food and sufficient energy while maintaining a constant body temperature, which is essential for winter survival.

*Firmicutes* and *Bacteroidota* are the dominant bacteria in gut microbiota of goitered gazelles, and this is common in other herbivores (Ilina et al., 2021; Liu et al., 2021b). *Firmicutes* are involved in the digestion of cellulose and energy metabolism and are indispensable to herbivores (Chen et al., 2017; Zhao et al., 2018). In this study, the relative abundance of *Firmicutes* was significantly higher in winter, indicating that *Firmicutes* may be important in the winter adaptation of goitered gazelles. *Christensenellaceae* bacteria are associated with the body mass index (BMI) of the host and negatively correlated with visceral fat mass and trunk fat (Waters and Ley, 2019). The relative abundance of *Christensenellaceae* was significantly higher in the winter, and this suggested that the visceral fat mass and trunk fat are lower in the winter than in the summer. Because *Christensenellaceae* are associated with healthy glucose metabolism (Waters and Ley, 2019), they could promote the glucose conversion into energy to aid winter survival of goitered gazelles. The relative abundance of *Bacteroides* is also significantly higher in the winter, and this may be related to carbohydrate metabolism to enhance the nutrient utilization of the goitered gazelles (Li et al., 2017b). The relative abundance of “Metabolism” functions is significantly higher in the winter to maintain the basal metabolism of the goitered gazelles. To metabolize high-fat food in the summer, the relative abundance of *Rikenellaceae* was significantly higher in the summer (Daniel et al., 2014). Goitered gazelles in Keke Town can therefore use gut microbiota to enhance their seasonal adaptations, especially in the winter.

The ratio of *Firmicutes*/*Bacteroidota* can reflect the nutrition status of the host. When food has a low carbohydrate content, the ratio decreases (De Filippo et al., 2010). However, in goitered gazelles, the gut microbiota ratios showed no significant differences between winter and summer. This means there is no significant seasonal variation in the nutritional status of goitered gazelles at Keke Town, and there is no significant decline in their nutritional status during the winter. We speculated that the goitered gazelle maintains the seasonal balance of nutrition by regulating the relative abundance of different bacteria in the gut.

### Effects of Seasonal Dietary Changes on the Gut Microbiota of Goitered Gazelles

Diet is the main factor that affects gut microbial diversity, and seasonal dietary changes contribute to the seasonal changes of gut microbiota (Zhang et al., 2014; Li et al., 2019a; Guo et al., 2021; Huang and Liao, 2021). In the winter, there are 17 plant species consumed by goitered gazelles while 30 plant species are consumed in the summer (Xu et al., 2008). The α-diversity of gut microbiota in the winter was higher than in the summer. A healthy, diverse diet promotes diverse gut microbiota (Zmora et al., 2018). However, our result was not consistent with this general conclusion. There are two explanations for this phenomenon. The first one is that the seasonal dietary changes of goitered gazelles in Keke Town are different from those of Xinjiang. Maybe even the opposite. The second one is that it is a compensatory measure. More bacteria with more functional genes that can improve nutrition status with a large number of microorganisms in the winter and maintain the nutritional balance between winter and summer. For example, the relative abundance of “Metabolism” significant higher in the winter, it may exist to maintain stable metabolic levels. However, the seasonal dietary changes and the specific compensation mechanism all need further study.

### Effects of Soil Microbiota on the Gut Microbiota of Goitered Gazelles

No previous studies have evaluated the contribution of soil microbiota to herbivores in the Qinghai-Tibetan plateau. We found that the gut microbiota of goitered gazelles had more microbiota from soil than zokors that live underground (Liu et al., 2021a). This suggests that goitered gazelles contact the soil when feeding and obtain microorganisms from the soil. The gut microbiota of goitered gazelle is clearly affected by soil microbiota. There were significantly more bacteria in the gut that originated from soil microbiota in the summer than in the winter. This indicated that goitered gazelles have more contact with soil or lick more soil in the summer, but this is not consistent with the results of higher diversity of gut microbiota in the winter. We believe that the intake mass of soil microorganisms is the result of voluntary selection by goitered gazelles. *Firmicutes* in the soil showed no significant differences between winter and summer, which is different from what happens in the gut. However, the relative abundance of *Bacteroidota* showed a significant increase in soil in the summer, but no significant differences were seen in the gut microbiota of goitered gazelles between winter and summer. It is possible that *Firmicutes* and *Bacteroidota* in soil have no seasonal effect on the gut microbiota of goitered gazelles, and these two dominant bacteria are affected by other factors. However, soil microbiota effects on goitered gazelles at Keke Town need further study.

### Seasonal Variations of Ecological Assembly Processes of Microbiota Communities in the Gut and Soil

The seasonal variations of ecological assembly processes of microbiota communities in the gut and soil are the parts of the contributions that elucidate the seasonal changes of microbiota in the gut and soil. We found that seasonal variations have an important influence on the assembly of microbiota in the gut of goitered gazelles and the soil by affecting the balance between deterministic and stochastic processes. The ecological processes between fecal microbiota and soil microbiota showed significant differences, and they were dominated by stochastic processes and deterministic processes, respectively.

Gut microbiota of goitered gazelles changes rapidly to maintain homeostasis within the intestinal environment. These changes include reproduction, death and exchanges of a large number of microorganisms, resulting in the dominance of the stochastic process. The soil environments on the Qaidam Basin are relatively stable, and the changes in ground temperature and precipitation are relatively slow (Shen, 1998; Zeng et al., 2020). Thus, the soil microbiota remains in a relatively dynamic balance. However, the soil microbiota may be exposed to relatively high physiological stress, including high altitude, cold and drought (Shen, 1998). Therefore, they are more competitive, resulting in the dominance of deterministic processes.

The seasonal variation of fecal microbiota explained by stochastic processes decreased from 50.6% in the winter to 31% in the summer. The same phenomena also appeared in the seasonal changes of soil microbiota, which decreased from 43.1% in the winter to 41.1% in the summer. One possible explanation is related to the seasonal variations of environments. In the summer, goitered gazelles consume more plant species, and they can obtain better nutrition in the summer than in the winter (Xu et al., 2008). This situation is beneficial for the growth and reproduction of gut microbiota, which would increase the physiological stress of gut microbiota of goitered gazelles. The average temperature, precipitation and sunshine hours all increase in the summer (Xu et al., 2020), which is beneficial to the growth and reproduction of soil microbiota. These conditions would also increase the physiological stress of soil microbiota. Lower physiological stress may let microbiota both in gut and soil grow and reproduce freely (Mo et al., 2021); therefore, the influences of stochastic processes are relatively decreased in the summer.

Due to different environmental factors, the dominant ecological processes and seasonal variation of microbiota in the gut and soil were different. The microbial seasonal changes of ecological assembly processes in gut and soil were the result of adaptation to the seasonal changes of the external environment. Although soil microbiota contributed a relatively large proportion to the gut microbial composition of the goitered gazelle, the ecological assembly processes of the microbiota between gut and soil changed obviously due to the huge difference in environments. The host can filter and select certain microbes from the external environment (Kohl, 2020). Therefore, in this study, the soil microbiota may not have a decisive effect on the seasonal variation of gut microbial diversity in goitered gazelles. Filtering and the gut environment of goitered gazelles may be the prior factors for the seasonal variation in the gut microbiota to the soil microbiota.

## Conclusion

The gut microbial diversity and functions showed significant seasonal differences in goitered gazelles. Goitered gazelle adapted to the winter environment by increasing the relative abundance of *Firmicutes*, *Christensenellaceae*, and *Bacteroides* to improve energy utilization. The ratio of *Firmicutes*/*Bacteroidota* showed no significant nutritional differences in goitered gazelles between winter and summer. These are the adaptive strategies that gut microbiota helps the host maintain a nutritional balance between winter and summer. Soil microbiota contribute to the gut microbiota of goitered gazelles relatively more based on source tracking analysis, and the seasonal changes in soil microbiota can have an influence on the diversity of gut microbiota. It is possible that goitered gazelles lick the soil to obtain microorganisms to maintain homeostasis. Due to the difference in assembly processes and physiology stresses, the dominant ecological processes in the gut are stochastic, and in soil, they are deterministic. Soil microbiota does not have a decisive effect on the gut microbiota of goitered gazelles. Goitered gazelles on the Qinghai-Tibetan plateau have co-evolved with their gut microbiota to survive. Perhaps filtering and the gut environment of goitered gazelles are the prior factors for the seasonal variation in the gut microbiota to the soil microbiota.

These results provide information useful for the conservation of goitered gazelles and also contribute to the analysis of environmental effects on gut microbiota. Understanding the seasonal adaptive strategies of goitered gazelles on the Qaidam Basin is beneficial to making specific conservation measures, and the goitered gazelle is a good example for studying the adaptations of animals in extreme environments.

## Data Availability Statement

The datasets presented in this study can be found in online repositories. The names of the repository/repositories and accession number(s) can be found in the article/Supplementary Material.

## Ethics Statement

The animal study was reviewed and approved by the Ethical Committee for Experimental Animals’ Welfare of Northwest Institute of Plateau Biology.

## Author Contributions

WQ and PS wrote the manuscript and analyzed the data. WQ contributed to sampling and laboratory experiments. SZ designed the study. All authors contributed to the article and approved the submitted version.

## Funding

This study was supported by The Open Project of State Key Laboratory of Plateau Ecology and Agriculture, Qinghai University (2020-ZZ-08).

## Conflict of Interest

The authors declare that the research was conducted in the absence of any commercial or financial relationships that could be construed as a potential conflict of interest.

## Publisher’s Note

All claims expressed in this article are solely those of the authors and do not necessarily represent those of their affiliated organizations, or those of the publisher, the editors and the reviewers. Any product that may be evaluated in this article, or claim that may be made by its manufacturer, is not guaranteed or endorsed by the publisher.

## Footnotes

^1^ https://www.arb-silva.de/fileadmin/silva_databases/release_138/Exports/SILVA_138_SSURef_NR99_tax_silva.fasta.gz

^2^ https://forum.qiime2.org/t/processing-filtering-and-evaluating-the-silva-database-and-other-reference-sequence-data-with-rescript/15494

^3^ https://cran.r-project.org/web/packages/minpack.lm/index.html

^4^ https://github.com/GotelliLab/EcoSimR/
