# Supplementary material for: Seasonal and Soil Microbiota Effects on the Adaptive Strategies of Wild Goitered Gazelles Based on the Gut Microbiota
Source: Front Microbiol. 2022 Jul 4;13:918090. doi: 10.3389/fmicb.2022.918090 (PMC9289685; doi:10.3389/fmicb.2022.918090)
Supplement: Supplementary file 1 [file Data_Sheet_1.docx]

Appendix 1: Results of the PERMANOVA analysis of the gut microbial diversities between winter and summer.

| Name | Df | Sums Of Sqs | MeanSqs | F.Models | R2 | Pr(>F) |
| --- | --- | --- | --- | --- | --- | --- |
| Feces | 1 | 2.633677 | 2.633677 | 10.96542 | 0.126089 | 0.001 |
| Residuals | 76 | 18.25371 | 0.24018 | - | 0.873911 | - |
| Total | 77 | 20.88738 | - | - | 1 | - |

Appendix 2: Results of the PERMANOVA analysis of the soil microbial diversities between winter and summer.

| Name | Df | Sums Of Sqs | MeanSqs | F.Models | R2 | Pr(>F) |
| --- | --- | --- | --- | --- | --- | --- |
| Soil | 1 | 1.442082 | 1.442082 | 5.288964 | 0.305916 | 0.004 |
| Residuals | 12 | 3.271905 | 0.272659 | - | 0.694084 | - |
| Total | 13 | 4.713988 | - | - | 1 | - |
